# Identification of sweetpotato virus disease-infected leaves from field images using deep learning

**DOI:** 10.3389/fpls.2024.1456713

**Published:** 2024-11-08

**Authors:** Ziyu Ding, Fanguo Zeng, Haifeng Li, Jianyu Zheng, Junzhi Chen, Biao Chen, Wenshan Zhong, Xuantian Li, Zhangying Wang, Lifei Huang, Xuejun Yue

**Affiliations:** ^1^ College of Electronic Engineering (College of Artificial Intelligence), South China Agricultural University, Guangzhou, China; ^2^ Guangdong Provincial Key Laboratory of Crops Genetics and Improvement, Crop Research Institute, Guangdong Academy of Agricultural Sciences, Guangzhou, Guangdong, China; ^3^ National Key Laboratory of Green Pesticides, South China Agricultural University, Guangzhou, China

**Keywords:** sweetpotato, virus disease, deep learning, RGB image, semantic segmentation

## Abstract

**Introduction:**

Sweetpotato virus disease (SPVD) is widespread and causes significant economic losses. Current diagnostic methods are either costly or labor-intensive, limiting both efficiency and scalability.

**Methods:**

The segmentation algorithm proposed in this study can rapidly and accurately identify SPVD lesions from field-captured photos of sweetpotato leaves. Two custom datasets, DS-1 and DS-2, are utilized, containing meticulously annotated images of sweetpotato leaves affected by SPVD. DS-1 is used for training, validation, and testing the model, while DS-2 is exclusively employed to validate the model’s reliability. This study employs a deep learning-based semantic segmentation network, DeepLabV3+, integrated with an Attention Pyramid Fusion (APF) module. The APF module combines a channel attention mechanism with multi-scale feature fusion to enhance the model’s performance in disease pixel segmentation. Additionally, a novel data augmentation technique is utilized to improve recognition accuracy in the edge background areas of real large images, addressing issues of poor segmentation precision in these regions. Transfer learning is applied to enhance the model’s generalization capabilities.

**Results:**

The experimental results indicate that the model, with 62.57M parameters and 253.92 Giga Floating Point Operations Per Second (GFLOPs), achieves a mean Intersection over Union (mIoU) of 94.63% and a mean accuracy (mAcc) of 96.99% on the DS-1 test set, and an mIoU of 78.59% and an mAcc of 79.47% on the DS-2 dataset.

**Discussion:**

Ablation studies confirm the effectiveness of the proposed data augmentation and APF methods, while comparative experiments demonstrate the model’s superiority across various metrics. The proposed method also exhibits excellent detection results in simulated scenarios. In summary, this study successfully deploys a deep learning framework to segment SPVD lesions from field images of sweetpotato foliage, which will contribute to the rapid and intelligent detection of sweetpotato diseases.

## 1 Introduction

Sweetpotato (*Ipomoea batatas* L.) is one of the top ten food crops in the world and an important source of nutrients for the human body (Lee et al., 2019). Currently, China has the largest sweetpotato planting area of about 6.6 million hm^2^ and an output of about 100 million tons, accounting for roughly 80% of the world’s total output annually (Gai et al., 2016; Zhang et al., 2019). Sweetpotatoes are propagated through vines and tubers, making them especially vulnerable to viral diseases that can be transmitted from one generation to the next. This mode of transmission often leads to the rapid degradation of new sweetpotato varieties and significant yield losses (McGregor et al., 2009; Clark et al., 2012). The most serious of these viral diseases is sweetpotato virus disease (SPVD), which results from a symbiotic infection of the sweetpotato feathery mottle virus (SPFMV) and the sweetpotato chlorotic stunt virus (SPCSV) (Rännäli et al., 2009; Wanjala et al., 2020).

Since 2012, SPVD has rapidly spread across China, severely affecting key sweetpotato-growing regions. The disease has a devastating impact on sweetpotato production, causing yield losses of 90-100% (Karyeija et al., 1998). For instance, a major SPVD outbreak occurred in Zhanjiang City, Guangdong Province, in early 2015, where 1,257 hectares of sweetpotato fields were severely impacted, with an incidence rate exceeding 50% (Zhang et al., 2019). The prevalence of SPVD in China led to a restructuring of the sweetpotato market, making the production of virus-free seedlings the new standard. However, the lack of efficient virus detection technologies has hindered the supply and breeding of detoxified seedlings. In particular, there is an urgent need for simple and rapid detection methods for use at the grassroots level and for timely testing of seedling quality. Therefore, developing a reliable SPVD detection system is essential for managing the disease and supporting the breeding of virus-free sweetpotato seedlings.

Current research on SPVD has primarily focused on defense mechanisms, particularly virus detection and pathogenesis. The main detection techniques for SPVD include biological, serological, and molecular methods. Among these, enzyme-linked immunosorbent assay (ELISA) and PCR are the most widely used serological methods for SPVD detection (Wanjala et al., 2020; David et al., 2022). However, both ELISA and PCR are labor-intensive, expensive, and time-consuming. As China’s sweetpotato industry has expanded, the limitations of these methods have made it increasingly difficult to meet the growing demand for efficient SPVD detection. Thus, there is an urgent need for new, faster, and more efficient detection solutions, such as artificial intelligence (AI) and deep learning technologies, to overcome the shortcomings of manual and conventional SPVD identification methods.

The field of computer vision (CV) has achieved significant success across various applications, particularly in the area of plant disease detection (Srinivasu et al., 2024a; Patil and Manohar, 2022). Recent advancements in convolutional neural networks (CNNs) have revolutionized disease recognition, exemplified by their application in potato disease detection using RGB images, where high precision has been attained (Oppenheim et al., 2019). Further illustrating the efficacy of deep learning, researchers have successfully employed these techniques to identify northern leaf blight in corn, achieving notable accuracy in field conditions (DeChant et al., 2017).

Progress has been marked by a shift from basic classification tasks to more complex challenges, such as pinpointing infected areas and assessing disease severity (Kalaivani et al., 2020). For instance, Zhou et al. utilized an enhanced DeepLabV3+ model for efficient recognition of tea leaf diseases, where the integration of an attention mechanism notably improved the model’s sensitivity to subtle lesions (Zhou et al., 2024). Similarly, Yang et al. advanced the detection of rice blast by adopting a U-Net architecture combined with a multi-scale feature fusion strategy, thus improving detection accuracy (Yang et al., 2023). Additionally, Li et al. proposed a Transformer-based framework for the real-time monitoring of wheat stripe rust, showcasing the capacity of deep learning for timely disease detection in agricultural settings (Zhu et al., 2022). Moreover, the evaluation of disease incidence on cucumber leaves under natural conditions has been facilitated by the improved DeepLabV3+ network, further demonstrating the robustness of CV technologies in various crop contexts (Wang et al., 2021; Li et al., 2022).

Despite these advancements, the application of CV technology in sweetpotato disease detection remains underexplored. Current research primarily focuses on other crops such as potatoes, corn, tomatoes, rice, and wheat, leaving a gap in knowledge regarding sweetpotato diseases. Although deep learning techniques have been utilized for counting sweetpotato leaves (Wang et al., 2023), their impact on disease management and detection in practical scenarios is limited. This is particularly critical given the lack of simple and rapid detection methods at the grassroots level. Therefore, timely and accurate detection of sweetpotato virus diseases is essential for effective disease management and agricultural sustainability.

Motivated by these technical gaps and the need for efficient SPVD detection, this study employs deep learning techniques to identify SPVD lesions on sweetpotato leaves. RGB photos of sweetpotato leaves from the field that showed typical SPVD symptoms at the “branching tuber stage” were gathered, and the DeepLabV3+ deep learning segmentation model was utilized to precisely identify the affected regions. Data enhancement, transfer learning, and a proposed APF module were implemented to enhance the recognition performance. The model’s SPVD detection efficacy was evaluated on two datasets (DS-1 and DS-2). The proposed system aims to provide growers with an efficient, easy-to-apply, and affordable solution for SPVD diagnosis, facilitating SPVD management and reducing economic losses caused by the disease.

The main contributions of this study are as follows:

- A data augmentation method is proposed to process edge background areas through a specific strategy, which significantly improves the model’s recognition accuracy of edge background areas in real large images.
- The proposed attention pyramid fusion (APF) significantly enhances the model’s features at different scales by introducing a channel attention module and fusing multi-scale features, thereby enhancing its performance in disease pixel segmentation.
- Through ablation studies and comparative experiments on a self-made SPVD dataset, the effectiveness of data augmentation and APF methods are verified, and excellent detection results are shown in simulated scenarios.

The rest of this paper is organized as follows: Section 2 introduces the relevant materials and methods. The materials include the obtained datasets and how to process them, while the methodology is a description of the details of the proposed improved deeplabv3+. Section 3 analyzes the experimental results and discusses the impact of the network module. Section 4 discusses and makes suggestions for future research.

## 2 Materials and methods

### 2.1 SPVD dataset collection

**Figure 1:**
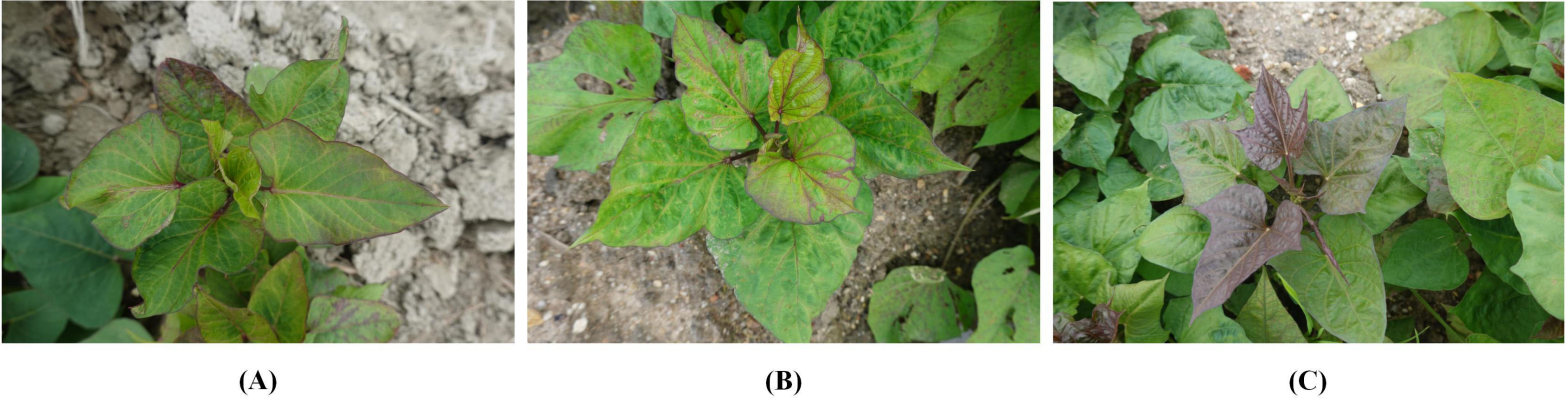
Example images in the SPVD dataset. Typical symptoms of SPVD in the late stage of infection can be identified by yellowed, veined, deformed leaves, and dwarfed plants. **(A)**, “Fragrant Pink Potato”; the top leaves were green pointed heart-shaped, and the mature leaves were green heart-shaped. **(B)**, “Guangshu 22-18”; the top leaves were green pointed heart-shaped, and the mature leaves were green heart-shaped. **(C)**, “Guangshu 22-15”; both top and mature leaves were shallow split single notch-shaped, with the top leaves being purple and mature leaves being green.

Due to the lack of publicly available datasets for studying SPVD, this study collected and constructed two distinct RGB datasets from field environments, referred to as DS-1 and DS-2. To ensure the representativeness of DS-1, data were gathered from a 100-acre farmland near Maoming City, Guangdong Province, China, where the local variety “Sweet Fragrant Potato” had been planted for 40-50 days, corresponding to the “Branching Tuber Stage.” The top leaves of the plants were green and pointed heart-shaped, while the mature leaves were green and heart-shaped (**Figure 1A**). Suspected infected plants exhibited typical symptoms of SPVD, such as stunted growth, twisted leaf veins, chlorosis of leaves and petiole leaflets, as well as yellowing of leaf veins. Experts in plant diseases initially classified these symptoms as late-stage SPVD infection, which is the maximum degree of severity. This made it easier to gather photographic evidence and build the study’s model.

In January 2022, 216 suspected infected plants were photographed using a handheld SONY DSC-RX100M6 digital camera (Sony Group Corporation, Tokyo, Japan) with close-up and horizontal scanning perspectives under the guidance of a photographer. To obtain detailed information about SPVD detection, images with less obvious symptoms were removed, and 300 high-resolution RGB images in JPEG format (5472×3648 pixels) were selected. Additionally, in September 2023, virus disease symptoms were observed in the sweetpotato resource nursery (45 acres)in Guangzhou, China, and plant leaves with typical SPVD symptoms were selected. Considering the reliance of computer vision technology on visual features, two varieties with appearances similar to “Sweet Fragrant Potato” in DS-1 were chosen, and 12 high-resolution images were captured to constitute DS-2. Among them, the variety ‘Guangshu 22-18’ comprised 8 plants, characterized by green pointed heart-shaped top leaves and green heart-shaped mature leaves (**Figure 1B**); the variety ‘Guangshu 22-15’ comprised 4 plants. The top leaf color of the plant was purple, and the leaf shape was shallow split single notch, while the mature leaf color was green, and the leaf shape was shallow split single notch (**Figure 1C**). The variety “Guangshu 22-18” was highly similar to “Sweet Fragrant Potato” in terms of leaf color and shape, while the variety “Guangshu 22-15” had slight differences in leaf shape and completely different top leaf color. These sweetpotatoes had been planted for 50-60 days (“Branching tuber stage “), and experts preliminarily identified them as being in the late stage of SPVD infection. In summary, the DS-1 dataset is used to train, validate, and test the performance of the model. The DS-2 dataset is specifically used to test the generalization ability of the model. Statistical details are provided in **Table 1**. In Section 2.3, the DS-1 and DS-2 datasets were further preprocessed to ensure suitability for specific task requirements. Samples collected from both locations were promptly placed in ice chests, frozen rapidly in liquid nitrogen, and stored in a -80°C freezer for molecular biology identification purposes.

**Table 1:** SPVD dataset information.

Dataset	Subset	Resolution	Number of images
DS-1	Training	5472×3648	300
	Validation		
	Test-1		
DS-2	Test-2	5472×3648	12

### 2.2 Materials and methods for virus identification

Materials: Plant RNA Kit, Omega Bio-Tek, USA; EasyScript OneStep gDNA Removal and cDNA Synthesis SuperMix Kit, TransGen Biotech Co., Ltd., China; 2×TaqMaster Mix (With Dye), APExBIO Technology LLC, USA; Primers synthesized by Sangon Biotech (Shanghai) Co., Ltd., China; Biometra TAdvanced PCR Instrument, Biometra GmbH, Germany; DYY-6C Electrophoresis Apparatus, Beijing Liuyi Biotechnology Co., Ltd., China; Tanon 4100 Gel Imaging System, Shanghai Tianeng Technology Co., Ltd., China.

The collected sweetpotato plant leaves were ground into powder in liquid nitrogen. The total RNA was extracted using a Total RNA Extraction Kit, followed by cDNA synthesis using a reverse transcription kit. The reverse transcription reaction system was 20 μL. For the reverse transcription, 1 ug of the RNA template was denatured at 65°C for 5 minutes and then placed in an ice bath for 2 minutes. Next, 1μL of Anchored Oligo (dT)^18^ Primer (0.5 μg/μL), 1μL of EasyScript^®^ RT/RI Enzyme Mix, and 10 μL of 2×ES Reaction Mix were added and topped up to 20 μL with RNase-Free H_2_O. The process was maintained at 42°C for 15 minutes and then 85°C for 5 seconds to complete the reverse transcription reaction. RT-PCR amplification was performed using RNA virus-specific primers (primer sequences listed in **Table 2**) (Li et al., 2012; Pan et al., 2013). The RT-PCR reaction system (25 μL) included 2 μL of synthesized cDNA, 12.5 μL of 2×Taq Master Mix (With Dye), 1μL each of upstream and downstream primers (10 μmol/L), and 8.5 μL of ddH_2_O. The amplification conditions were as follows: 94°C for 2 minutes; 30 cycles of 94°C for 30 seconds, annealing at 45°C (temperature specified in **Table 2**) for 45 seconds, and extension at 68°C for 1 minute; followed by a final extension at 68°C for 10 minutes. All assays included a negative control using water as a template. Next, the 5 μL amplified products were detected by 1% agarose gel electrophoresis and photographed by gel imager (Xinliang et al., 2022).

**Table 2:** Primers used in RT-PCR for the detection of sweetpotato viruses^a^.

Primer	Sequence^b^(5’-3’)	Product size/bp	Annealing Temperature/°C	Target virus
SPF-F1	GGATTAYGGTGTTGACGACACA	589	60	SPFMV
SPC-F1	GTGAGAAAYCTATGCGCTCTGTT	836		SPVC
SPG-F5	GTATGAAGACTCTCTGACAAATTTTG	1191		SPVG
SP2-F10	CGTACATTGAAAAGAGAAACAGGATA	369		SPV2
SPFCG-R2	TCGGGACTGAARGAYACGAATTTAA	–		SPVG,SPVC, SPFMV, SPV2
CSV1	AGTGGTGAYGTAATAGTCGGTGG	365	61.06	SPCSV
CSV2	GCTAACGATTCACADACAGACTTCA	365	59.24	

a F represents forward primer; R represents reverse primer.

b Y= C or T; R=A or G.

### 2.3 SPVD dataset process

**Figure 2:**
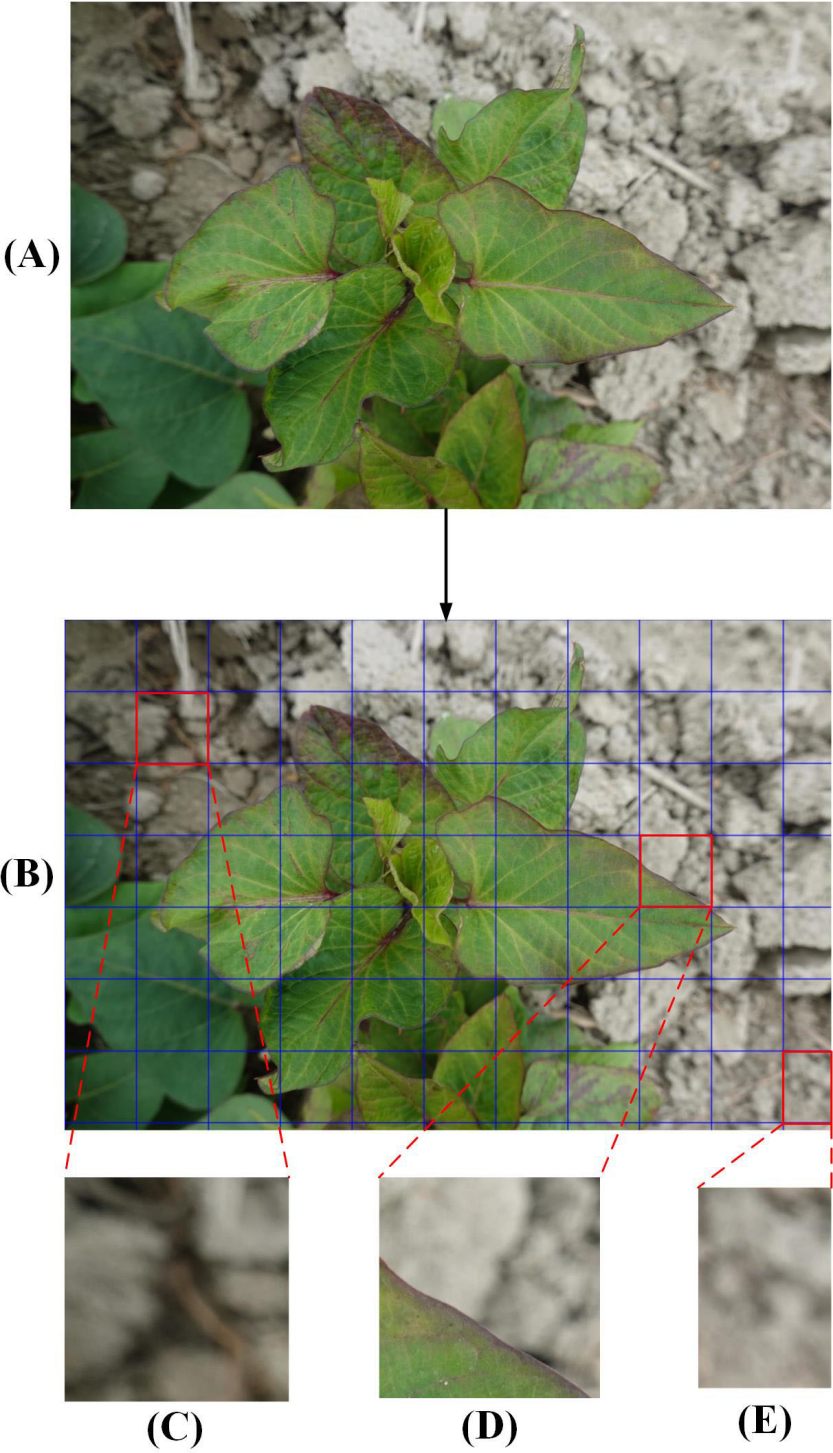
The process of dividing the original images into smaller images. **(A)**, A full-sized raw image in DS-1 before segmentation. **(B)**, Small-size image areas to be segmented. **(C)**, Original image background. **(D)**, Diseased leaf image. **(E)**, Cut residual image.

Due to the complexity of processing high-quality initial images and the limited GPU memory allocation, the original photos were clipped to 512 × 512 pixels (**Figures 2A, B**). The image background, such as the soil (**Figure 2C**) and the residual parts (**Figure 2E**) were discarded. Finally, 5,723 small-sized images containing only the diseased leaves portion (**Figure 2D**) were selected. Besides, images in DS-2 underwent the same processing, resulting in a total of 710 images sized at 512×512 pixels, which were not used in the model training process rather were only used to verify the model generalization ability and reliability.

**Figure 3:**
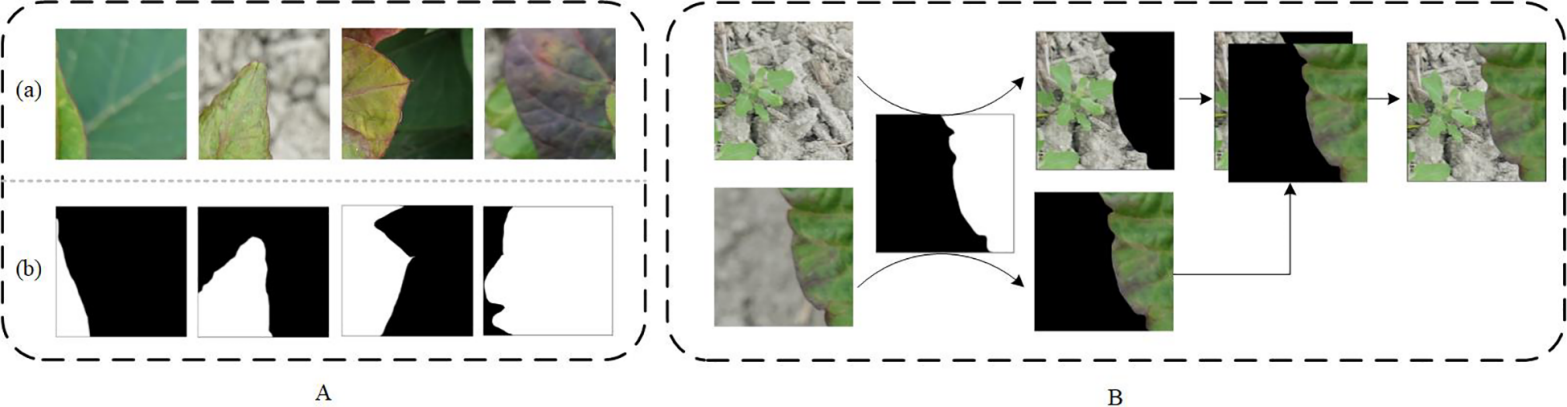
Data annotation and augmentation. **(A)**, Small-sized images and annotated images. **(B)**, The flowchart for making a composite image.

**Figure 4:**
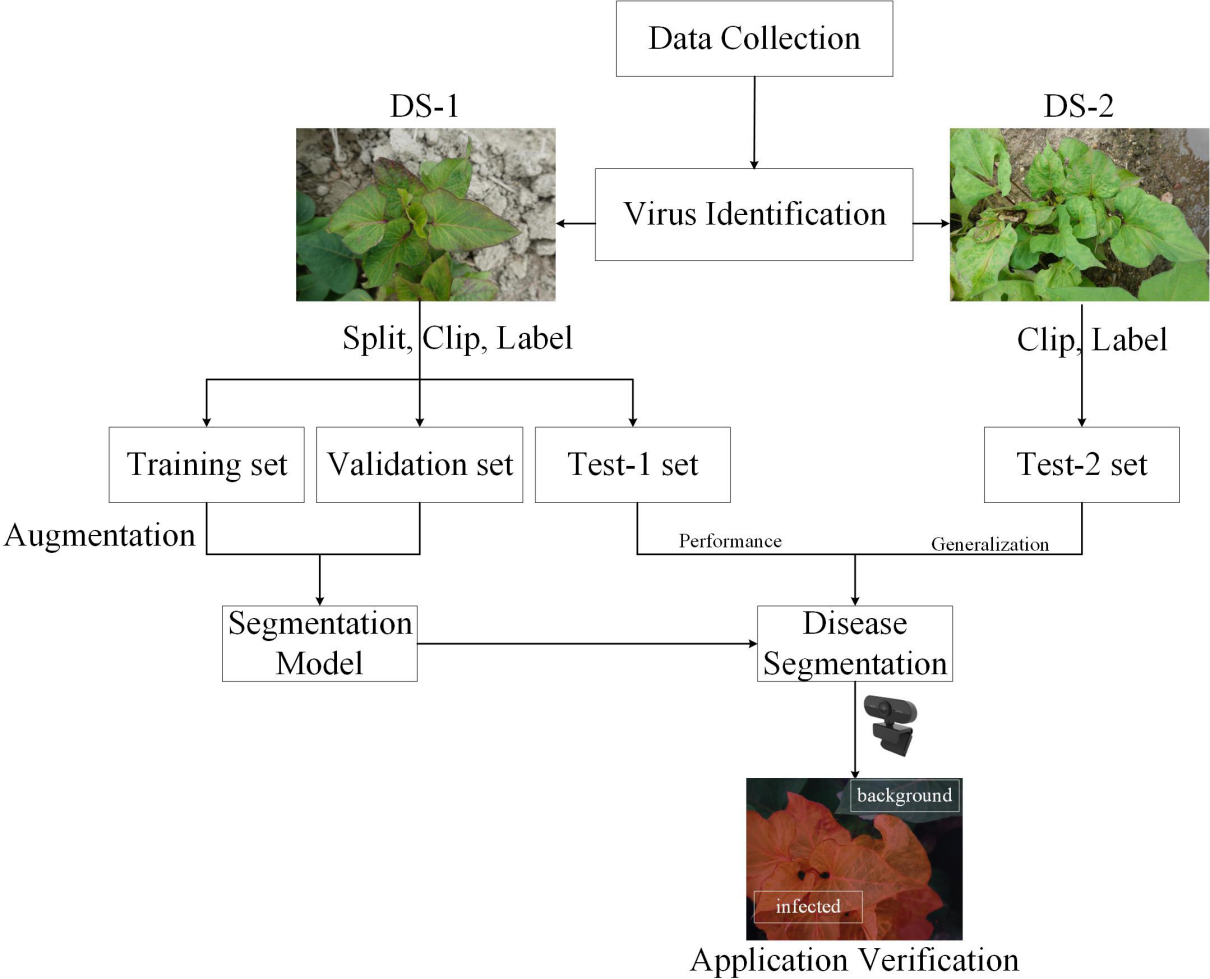
Overall flowchart of the methodology.

After the original image was segmented, the DS-1 and DS-2 images were labeled as infected or background. The leaf-diseased area was marked with polygons using the open-source computer vision annotation tool (CVAT). The generated images were saved in PNG format with ground truth annotations (**Figure 3A**). The ground truth labels were then divided into training, verification, and test sets in the ratio 8.1.1 with 4578, 572, and 573 images, respectively. To avoid over-fitting, the image augmentation method (Zou et al., 2021) was adopted to enhance the image diversity. Briefly, the infected area was cut out using the label as the foreground and overlaid on the background image to create a composite image (**Figure 3B**). To obtain a richer background, 165 background, and 27 foreground images were selected, generating 4455 composite images. After supplementing the training set with the obtained composite images, 9033 images were obtained. In addition, the process is shown in **Figure 4**.

### 2.4 Deep learning algorithm

**Figure 5:**
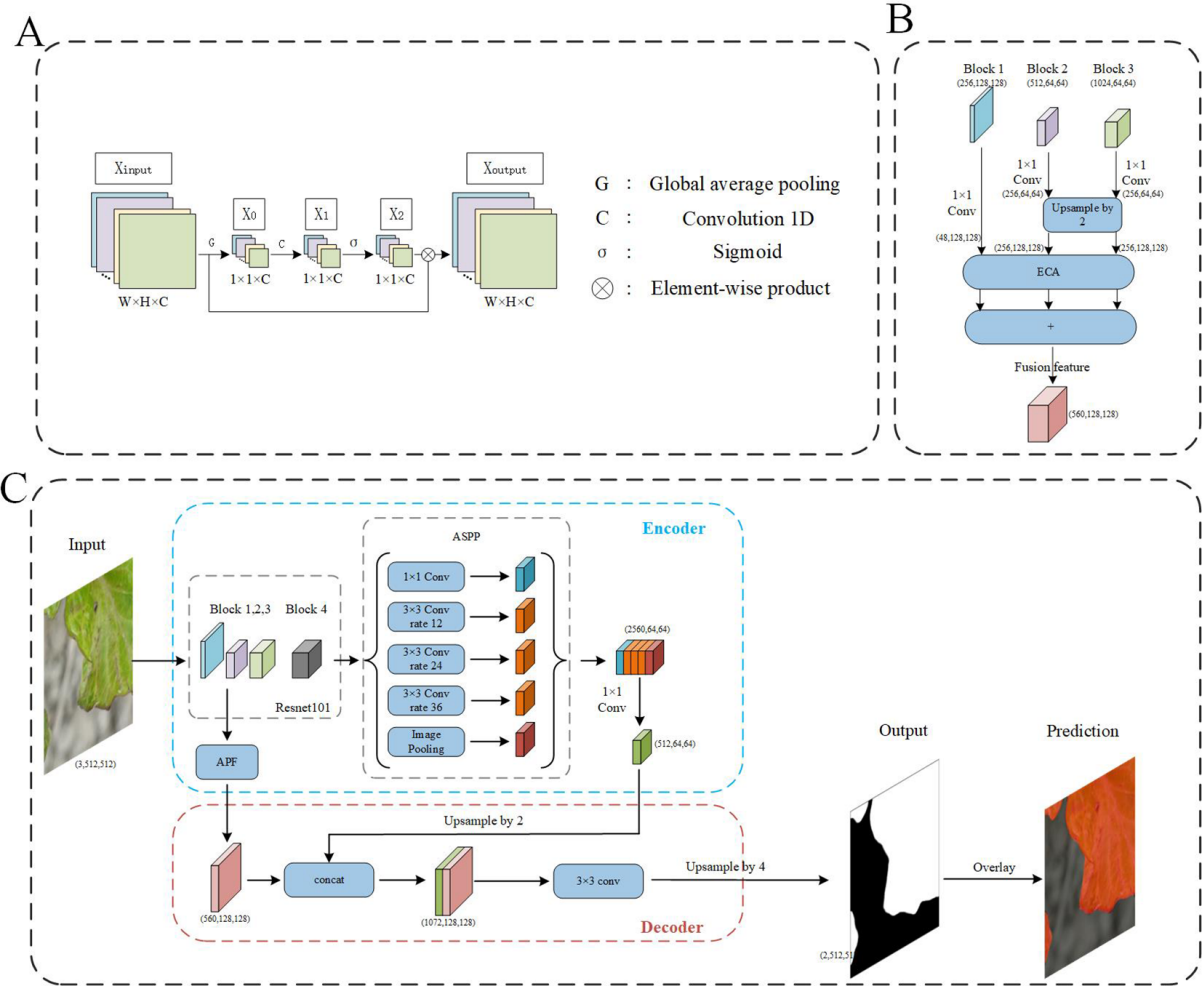
The flowchart of model. **(A)** The flowchart of ECA module. **(B)** The flowchart of APF module, **(C)** The flowchart of the segmentation network from small-sized images to prediction outputs.

The Efficient Channel Attention (ECA) module is a local cross-channel interaction strategy without dimensionality reduction (Wang et al., 2020), which can be effectively implemented by one-dimensional (1D) convolution, and is an effective channel attention learning method (**Figure 5A**). It is a lightweight general-purpose module that can be easily embedded into any CNN framework to achieve end-to-end training and significantly improve network performance, and its implementation process is shown in (**Figure 5B**). This method first uses global average pooling (GAP) to obtain X_0_ for each feature channel of the input feature map X_input_, then uses 1D convolution to capture local cross-channel interaction information to obtain X_1_, and then uses the Sigmoid function to generate channel weights X_2_, to get normalized weights between 0 and 1. Finally, the original feature image X_input_ with a matrix size of H×W×C is multiplied by the weight generated by the Sigmoid function to obtain a new feature image X_output_.

ResNet101 is a deep convolutional neural network architecture that introduces skip connections, allowing gradients to flow directly from later layers back to earlier layers during backpropagation (Zhang, 2022). The architecture typically serves as a backbone network for feature extraction and is composed of four stages, each containing multiple residual blocks. These stages serve as input features for downstream tasks, denoted as Block1, Block2, Block3, and Block4, with corresponding strides of (1, 2, 1, 1). A stride of 1 indicates that the output feature map size remains the same as the input feature map, without downsampling; a stride of 2 indicates that the output feature map size is half of the input feature map, indicating downsampling. After the second stage, the feature map size remains unchanged. Such a design helps maintain the resolution of the feature maps, which is particularly important for accurate segmentation in semantic segmentation tasks. Additionally, the activation function used is ReLU.

In the process of extracting image features using deep learning, the resolution of the image gradually decreases due to the continuous application of deep convolution operations, resulting in lower resolution deep features. This phenomenon is particularly detrimental to small objects within the image, leading to recognition errors. To address this issue, combining features from different levels during network training can significantly enhance the accuracy of multi-scale detection. Feature Pyramid Network (Lin et al., 2017) is an effective feature fusion method that combines feature maps from different layers to obtain feature representations that reflect semantic information at various scales. Different from FPN, the proposed attention pyramid fusion (APF) introduces a channel attention module on features of different scales, and then fuses the enhanced feature representations of different scales, further enhancing the representation ability of feature maps at different scales (**Figure 5B**, where (C, H, W) represents the feature map (channels, height, width). Specifically, Feature maps generated by Block1, Block2, and Block3 of the DeepLab v3+ backbone network ResNet101 are fused. These feature maps have sizes of 1/4, 1/8, and 1/8 of the input image, with initial channel numbers of 256, 512, and 1024, respectively. In APF, a 1×1 convolution is first applied to the feature maps of Block1, Block2, and Block3 for dimensionality reduction, optimizing computational efficiency and feature representation. Specifically, the number of channels in Block1’s feature map is reduced from 256 to 48, Block2’s feature map from 512 to 256, and Block3’s feature map from 1024 to 256. To unify the sizes of the feature maps, the feature maps of Block2 and Block3 are upsampled, increasing their sizes from 1/8 to 1/4 to match the size of Block1’s feature map. Finally, these three processed feature maps are combined to obtain the fused feature map. This fused feature map not only contains feature information from three different levels but also possesses richer semantic and spatial information due to the introduction of the channel attention module, significantly enhancing the performance of the DeepLab v3+ network in image segmentation tasks.

The DeepLabV3+ used in this article is one of the most popular semantic segmentation networks (Chen et al., 2018), which consists of two parts: encoder and decoder, and uses depthwise separable convolution (Chollet, 2017), which is more efficient. The encoder module reduces the spatial resolution and captures semantic information, and the decoder module recovers the spatial information and leads to a cleaner segmentation. The flow chart of the algorithm is shown in **Figure 5**, where (C, H, W) represents the feature map (channels, height, width). First, input the 512×512 pixels picture containing part of the diseased leaves into the backbone network ResNet101 in the encoder, which can be used to extract the diseased features, in which the representation information is extracted in the Block 1,2,3, and the semantic information is extracted in the Block4 (1024 channels and 1/8 input size). The Atrous Spatial Pyramid Pooling (ASPP) (Chen et al., 2017) mechanism with different atrous rates of atrous convolution is used in DeepLabV3+. Since the diseased area in those small images is generally larger, a higher hole rate (rate = 12, 24, 36) is used compared to the original network (rate = 6, 12, 18). At the same time, ASPP also contains a 1×1 dilated convolution and an image pooling layer. After splicing the multi-scale feature maps, use 1×1 atrous convolution to fuse information and reduce dimensions. The processed feature map is then input to the decoder module. The output of the encoder module is upsampled by a factor of 2 and then fused with the multi-scale enhanced features that have passed through the APF module. Afterwards, a 3×3 convolutional layer is used to extract features, followed by another simple bilinear upsampling by a factor of 4. Finally, classify each pixel to get the binarized prediction result. According to the binarized prediction result, the diseased areas in the small-scale image can be marked in red, which can clearly show the diseased leaves. Moreover, to speed up the convergence process and improve the generalization ability of the model, a transfer learning strategy was adopted for the backbone ResNet101 parameters. Specifically, the backbone parameters trained on public datasets were used as the initial parameters of the network.

### 2.5 Model verification process

Model detection was verified under simulated conditions using a 480p Guke camera with a resolution of 640×480 pixels per frame. These frames were subsequently resized to 512×512 pixels and input into the model for processing to obtain 512×512 pixels binarized prediction result. According to the binarized prediction result, the diseased areas in the small-scale image were marked in red. Finally, the 512×512 pixels binarized prediction images were resized to 640×480 pixels and displayed in a small window.

### 2.6 Training procedure and performance evaluation

The system hardware consists of an Intel Core i9-10900X CPU, 32 GB of RAM, and an NVIDIA GeForce RTX 3090 GPU with 24 GB of VRAM. The software environment comprises Python 3.9, PyTorch 1.12.0, and a WSL system running on Windows 11. The optimizer employs the SGD algorithm with a weight decay factor of 0.0005 and a momentum factor of 0.9. The initial learning rate is set to 0.01 and varies according to the poly strategy. The batch size is set at 4, with a maximum of 20,000 iterations. The loss function is based on cross-entropy. The paper (Srinivasu et al., 2024b) is referenced, and some important parameters are presented in **Table 3**.

**Table 3:** Training parameters used in proposed model.

Training parameters	Values
Input Size	(512, 512)
Learning policy	Poly
Optimizer Type	SGD
Learning Rate	0.01
Momentum	0.9
Weight Decay	0.0005
Loss function	CrossEntropy
Batch size	4
Atrous rates	[12, 24, 36]

To demonstrate the enhanced capabilities of the proposed SPVD recognition model, a comprehensive comparative analysis of pioneering and state-of-the-art deep learning architectures is conducted. This includes: 1. Fully Convolutional Networks (FCN): Introduced by Long et al (Long et al., 2015), FCN revolutionizes semantic segmentation by enabling end-to-end learning without fully connected layers, making segmentation more efficient and accurate. 2. Pyramid Scene Parsing Network (PSPNet): Developed by Zhao et al (Zhao et al., 2017), PSPNet introduces the pyramid pooling module, effectively aggregating context information at different scales. This is crucial for distinguishing fine-grained categories in SPVD recognition. 3. Segmenter Series: Specifically, Segmenter ViT-B (Vision Transformer Base version) and Segmenter ViT-S (Vision Transformer Small version). These models showcase the potential of Transformer-based approaches in semantic segmentation by modeling long-range dependencies through self-attention mechanisms. 4. SETR ViT-L: The Segmentation Transformer with a Large Vision Transformer variant demonstrates the power of large-scale Vision Transformers in the field of semantic segmentation, particularly adept at handling wide spatial variations. 5. SegFormer MiT-B4: A combination of lightweight Transformer blocks and multi-scale feature representations, SegFormer efficiently integrates these components to maintain high precision while keeping computational complexity low.

These benchmark algorithms are sourced from the mmsegmentation library, an open-source semantic segmentation toolbox based on PyTorch. This library provides implementations of various semantic segmentation models and supports multiple mainstream segmentation frameworks. The library is highly extensible and includes many advanced technologies built-in, which facilitate the acceleration of the development and training processes.

The evaluation indicators cover the number of parameters (Params, M), number of floating-point operations (FLOPs, GFLOPs), mean intersection over union (mIoU, %), mean accuracy (mAcc, %), and the segmentation time (ST, ms). Among them, Params and FLOPs are statistically analyzed. ST is calculated by dividing the sum of processing time for all images in the test set by their total number. mIoU is the commonly used evaluation index in semantic segmentation methods, revealing the overlap degree between the predicted and actual areas. The mAcc was the ratio of the number of correctly predicted pixels to the total number of pixels, indicating the accuracy of the prediction results.

mIoU and mAcc are calculated using formulas (1) and (2) below, where k+1 represents the number of categories, which is set to 2 in this case, implying that pixels in each image are classified into two categories: SPVD-infected areas and the background. p_ij_ represents the number of pixels of category i predicted to be category j, p_ii_ indicates the number of pixels of category i predicted to be category i, and p_ji_ indicates the number of pixels of category j predicted to be class i.


(1) $$mIoU=\frac{1}{k+1}\sum_{i=0}^{k}\frac{p_{ii}}{\sum_{j=0}^{k}p_{ij}+\sum_{j=0}^{k}p_{ji}-p_{ii}}$$

(2) $$mAcc=\;\frac{1}{k+1}\;\sum_{i=0}^{k}\frac{p_{ii}}{\sum_{j=0}^{k}pij}$$

## 3 Results

### 3.1 RT-PCR detection results

**Figure 6:**
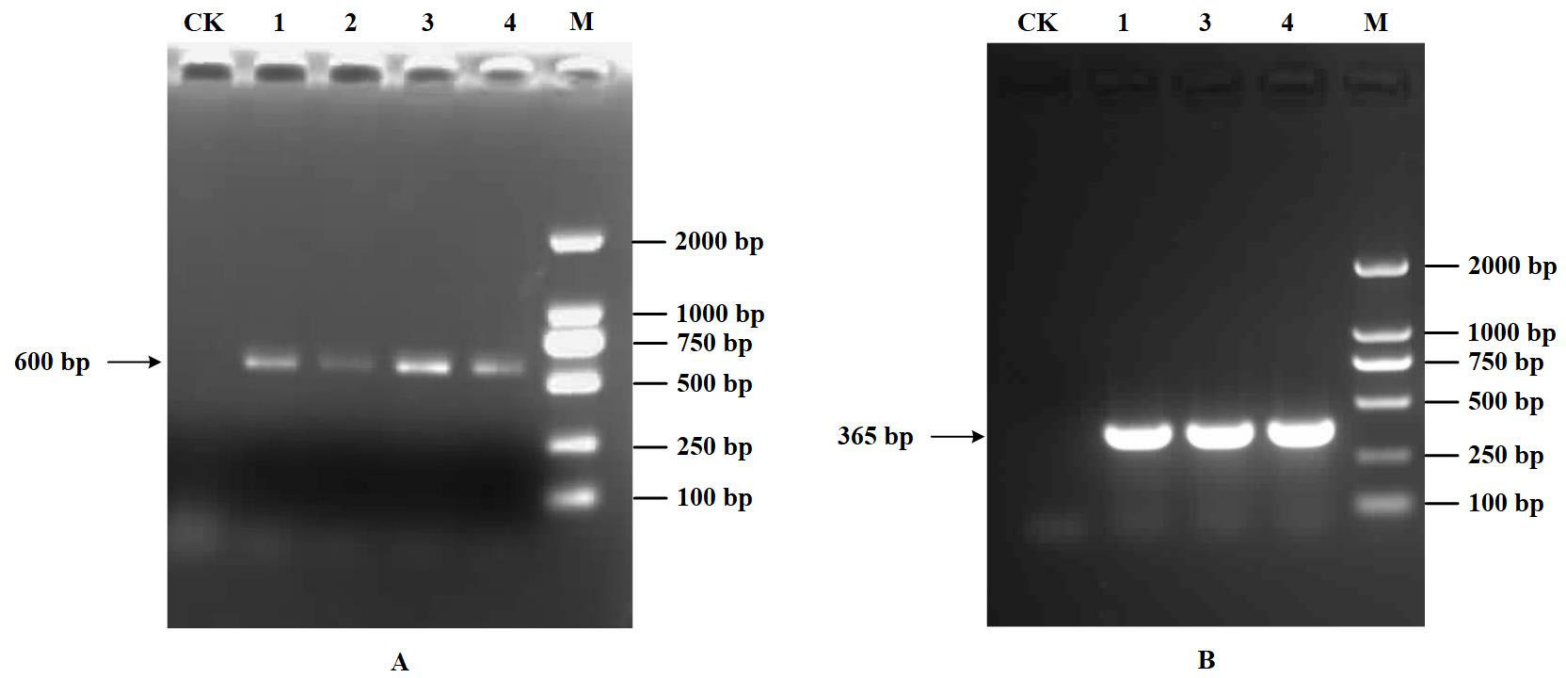
Detection results of RT-PCR. **(A)**, Detection results of RT-PCR. 1-4: SPFMV. **(B)**, Identification result of SPCSV. 1,3,4: SPCSV. M: DL2000 DNA maker, CK: healthy plant control.

Only a distinct band of approximately 600 bp within the 500-750 bp range was obtained based on the multiple detections of the four viruses, including SPVG, SPVC, SPFMV, and SPV2 using RT-PCR (**Figure 6A**). According to (Li et al., 2012), only 589 bp of SPFMV was amplified in four samples. Also, the presence of SPVG, SPVC, SPV2 was not detected. With the additional testing for the SPCSV virus, only a 365bp band was amplified (**Figure 6B**), confirming that the virus disease infecting the sweetpotato in the field was SPVD. Therefore, the diseased sweetpotato samples contained only two viruses, SPFMV and SPCSV. This, combined with the dwarfing and yellowing of sweetpotato plants in the field, indicates that the RGB data used for modeling analysis in this study is the sweetpotato data set infected with SPVD.

### 3.2 Ablation studies

The results from the ablation study revealed that the baseline model achieved a mIoU of 85.14% (**Table 4**). Compared to the baseline, the mIoU of baseline+DA was increased by 3.04% and mAcc by 0.45%. The mIoU of baseline+APF was 89.64%, and mAcc was 93.49%, which was better than the former. After incorporating APF into the baseline model with data augmentation (DA), the mIoU decreased by 0.12%. The performance improvement of baseline+TL was the most obvious, with mIoU and mAcc of 92.05 and 94.52%, respectively. Upon adding DA to the baseline with TL, mIoU and mAcc increased to 93.92% and 96.38%, respectively. After adding APF to the baseline model with TL, mIoU and mAcc increased by 2.58% and 2.47%, respectively.

**Table 4:** Performance comparison of different improvements on the network using the DS-1 test set^a^.

Methods	Baseline	DA	APF	TL	mIoU(%)	mAcc(%)
Baseline	√				85.14	93.39
Baseline + DA	√	√			88.18	93.84
Baseline + APF	√		√		89.64	93.49
Baseline + TL	√			√	92.05	94.52
Baseline + DA + APF	√	√	√		88.06	94.04
Baseline + DA + TL	√	√		√	93.92	96.38
Baseline + APF + TL	√		√	√	94.63	96.99
Baseline + DA + APF + TL **(Proposed)**	√	√	√	√	94.43	96.72

a Where √ indicates the strategy was used, blank means the strategy was not used. Baseline = DeepLabV3+ network with ResNet101 backbone, DA, data augmentation; APF, APF module; and TL, transfer learning; MIoU, mean intersection over union; MAcc, mean accuracy.

**Figure 7:**
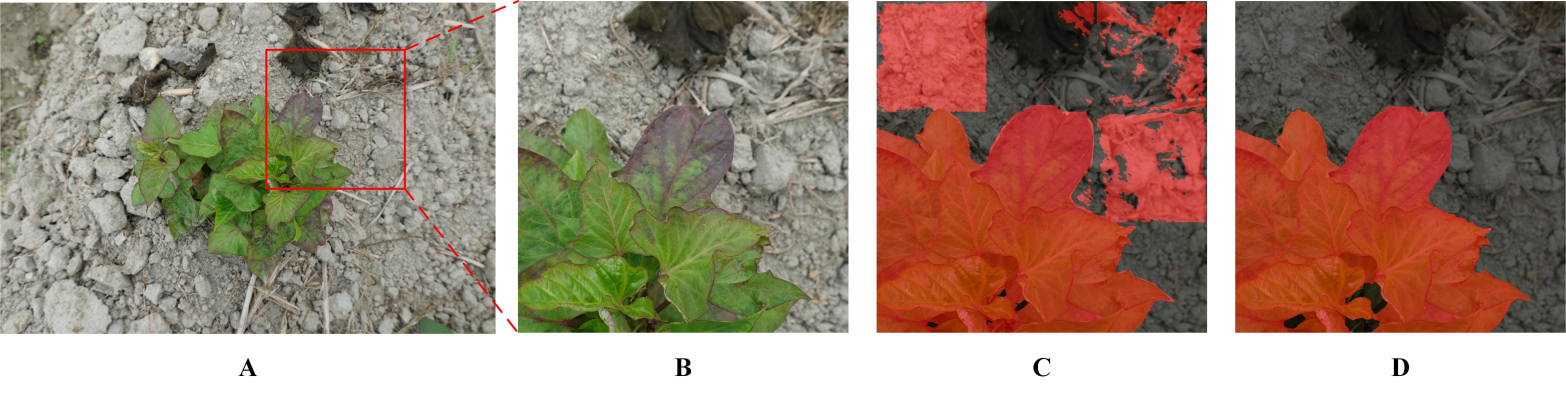
SPVD identification performance with and without DA. **(A)** A raw image. **(B)** The selected area on the raw image. **(C)** Recognition result based on baseline + APF + TL method. **(D)** Recognition result based on baseline + DA + APF + TL method (Proposed).

By comprehensively utilizing the APF and TL methods, the highest mIoU and mAcc scores achieved were 94.63 and 96.99%, respectively (a portion in **Figure 7A**). However, when the model was used to process pure background area, unsatisfactory performance was observed (**Figure 7B**). Specifically, the segmentation performance on the infected leaves was notably impressive, showcasing high accuracy. However, the recognition performance on the surrounding background was deficient, appearing as square red areas (**Figure 7C**). Although the mIoU and mAcc were slightly lower (0.2 and 0.27%, respectively) than the former after adding the DA, the overall effect was good (**Figure 7D**).

### 3.3 Comparison with other segmentation methods

**Figure 8:**
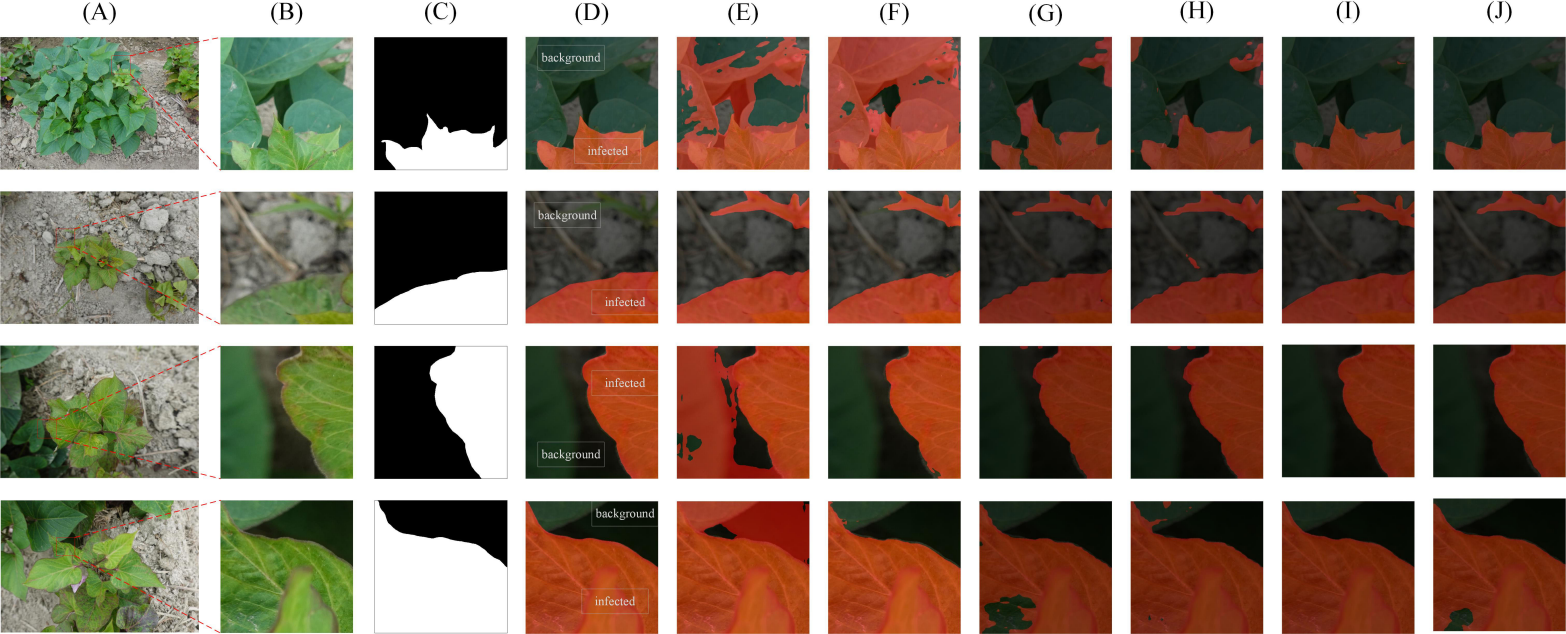
Segmentation results using different algorithms on DS-1. **(A)** Original images in DS-1 **(B)** The selected area **(C)** Ground truth **(D)** proposed algorithm **(E)** FCN **(F)** PSPNet **(G)** Segmenter ViT-B **(H)** Segmenter ViT-S **(I)** SegFormer MiT-B4 **(J)** SETR ViT-L.

**Figure 9:**
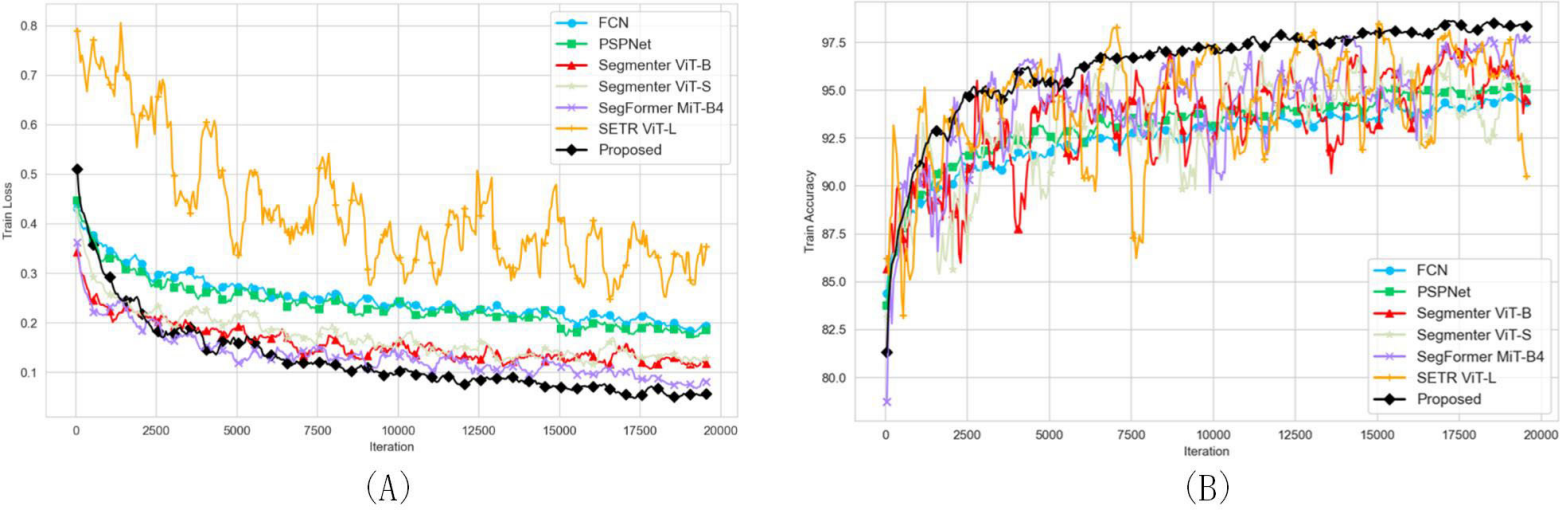
Training loss and accuracy curves for various semantic segmentation models. **(A)** Training Loss Curves **(B)** Training Accuracy Curves.

The mIoU and mAcc scores of the FCN, PSPNet and proposed network under the same conditions were higher than 85 and 90%, respectively, indicating that they can successfully complete the recognition challenge (**Table 5**). Segmenter-S is lighter in terms of parameter count and computational complexity (25.98 MB, 37.36 GFLOPs), making it suitable for deployment in resource-constrained environments. Although its performance (87.28% mIoU, 91.12% mAcc) is slightly lower than that of Segmenter-B (88.11% mIoU, 92.94% mAcc), it still demonstrates effective segmentation results, proving that lightweight models can achieve high segmentation accuracy while maintaining efficiency. SegFormer stands out with its relatively low parameter count (64.52 MB) and exceptional segmentation performance (91.27% mIoU, 94.45% mAcc). This is largely due to its innovative Transformer encoder design, which effectively leverages the self-attention mechanism to capture long-range dependencies in images while reducing computational complexity and memory consumption. SegFormer excels particularly in capturing detailed segmentation, accurately distinguishing between different object classes, as shown in **Figure 8**, where its segmentation results are often closer to ground truth values. While SETR (Segmentation Transformer) has the largest parameter count (309.33 MB) and relatively high computational complexity (367.21 GFLOPs), its segmentation performance (88.69% mIoU, 92.20% mAcc) did not reach the expected optimal levels. This may be due to the fact that the Transformer structure requires more data to train to fully leverage its advantages, and the training process might be more sensitive and unstable, as evidenced by the noticeable oscillations in the training curves. Although the proposed method shows slightly higher ST compared to other methods, it achieves the highest precision, exhibits stable training performance, and overall delivers the best performance. The training loss and accuracy curves of various semantic segmentation models are shown in **Figure 9**.

**Table 5:** Comparison results of different segmentation methods under enhanced data^a^.

Segmentation methods	Params(M)	FLOPs(GFLOPs)	MIoU(%)	MAcc (%)	ST(ms)
FCN	68.48	275.37	86.68	90.16	33.15
PSPNet	67.95	256.13	89.90	93.84	52
Segmenter-B	102.38	126.11	88.11	92.94	33.96
Segmenter-S	**25.98**	**37.36**	87.28	91.12	**26.21**
SegFormer	64.52	350.12	91.27	94.45	75.86
SETR	309.33	367.21	88.69	92.20	81.06
**Proposed**	62.57	253.92	**94.43**	**96.72**	50.61

a Params, parameters; FLOPs, floating-point operations; MIoU, mean intersection over union; MAcc, mean accuracy; ST, segmentation time. Numbers in bold indicate the best performance.

The comparison of segmentation details of different networks is shown in **Figure 8**, including the high-resolution original image (**Figure 8A**), its 512×512 pixel partial image (**Figure 8B**), and the corresponding ground truth labeled region (**Figure 8C**). The recognition results of FCN, PSPNet and our proposed method are shown in **Figures 8D–F**, respectively. The segmentation effects of the state-of-art models Segmenter ViT-B, Segmenter ViT-S, SegFormer MiT-B4 and SETR ViT-L are shown in **Figures 8G-J**, respectively. Among them, Segmenter ViT-B shows a certain accuracy in overall segmentation, but is slightly insufficient in processing details; Segmenter ViT-S shows a certain competitiveness in segmentation results while maintaining a relatively fast inference speed, but its segmentation details are slightly rough in complex backgrounds. SegFormer MiT-B4 has a very good segmentation effect. Its segmentation results are not only highly accurate, but also have smooth edges and well-processed details, which is close to our proposed method. Although SETR ViT-L has a large number of model parameters and high computational complexity, its segmentation effect does not fully meet expectations. The segmentation results of our proposed method are the best and closest to the ground truth.

### 3.4 Model generalization verification

**Figure 10:**
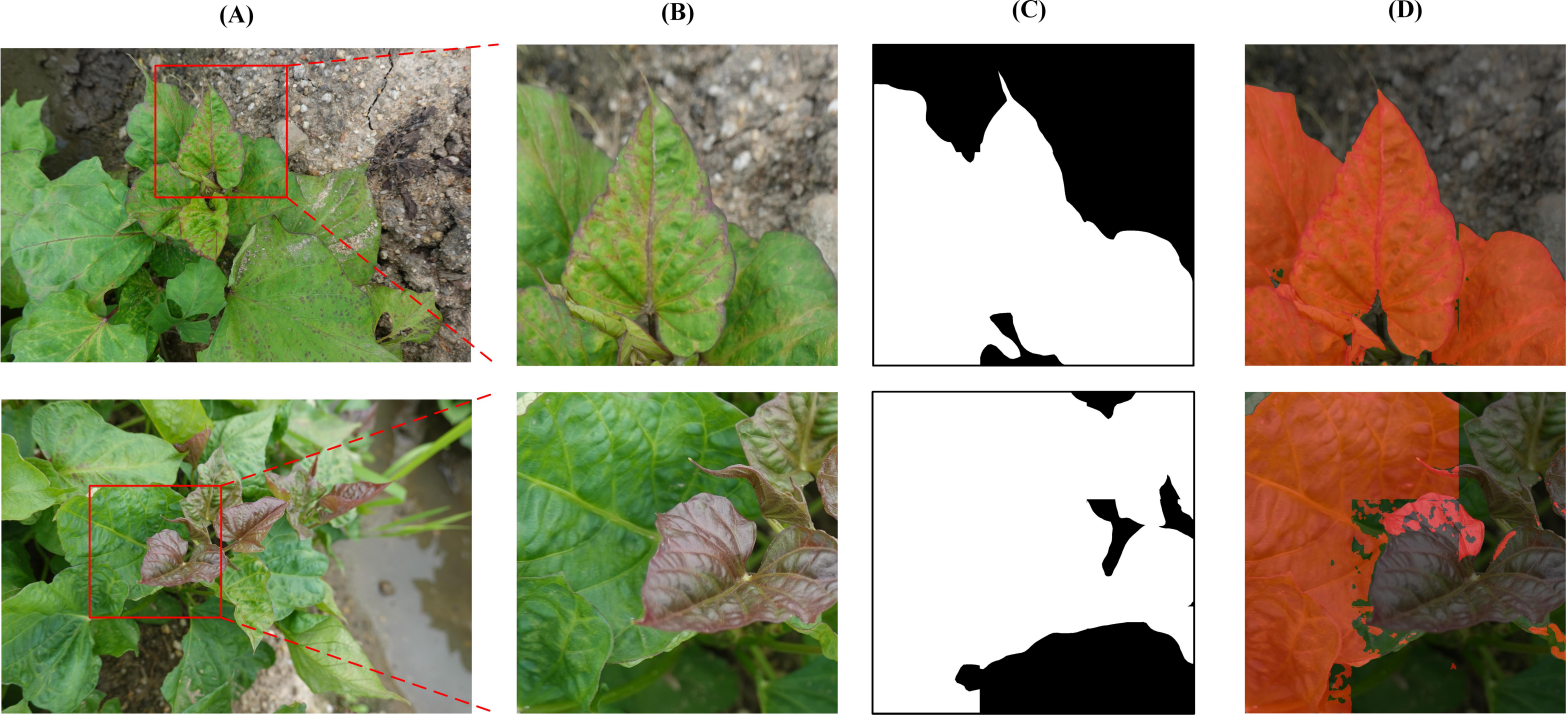
Segmentation results using proposed algorithm on DS-2. **(A)** Original images in DS-2. **(B)** The selected area **(C)** Ground truth **(D)** Detection results of proposed algorithm.

The model’s generalization is tested on DS-2, with the results summarized in **Table 6**. The mIoU and mAcc values are recorded as 78.59% and 79.47%, respectively, representing a decrease of 16.04% and 17.52%, respectively, compared to the DS-1 test set’s metrics of 94.63% and 96.99%. The segmentation results are shown in **Figure 10**. The model exhibits excellent recognition capability for disease features such as deformity and chlorosis on green top leaves and mature leaves, which are present in the DS-1 training set (**Figure 10**, first row). However, due to the lack of visual features of SPVD on purple top leaves in the DS-1 training set, the identification results for purple top leaves in DS-2 are poorer. (**Figure 10**, second row).

**Table 6:** Test results of the proposed method on DS-2^a^.

Segmentation method	MIoU(%)	MAcc (%)
**Proposed**	78.59	79.47

a MIoU, mean intersection over union; MAcc, mean accuracy.

### 3.5 Model application verification in simulation scenarios

**Figure 11:**
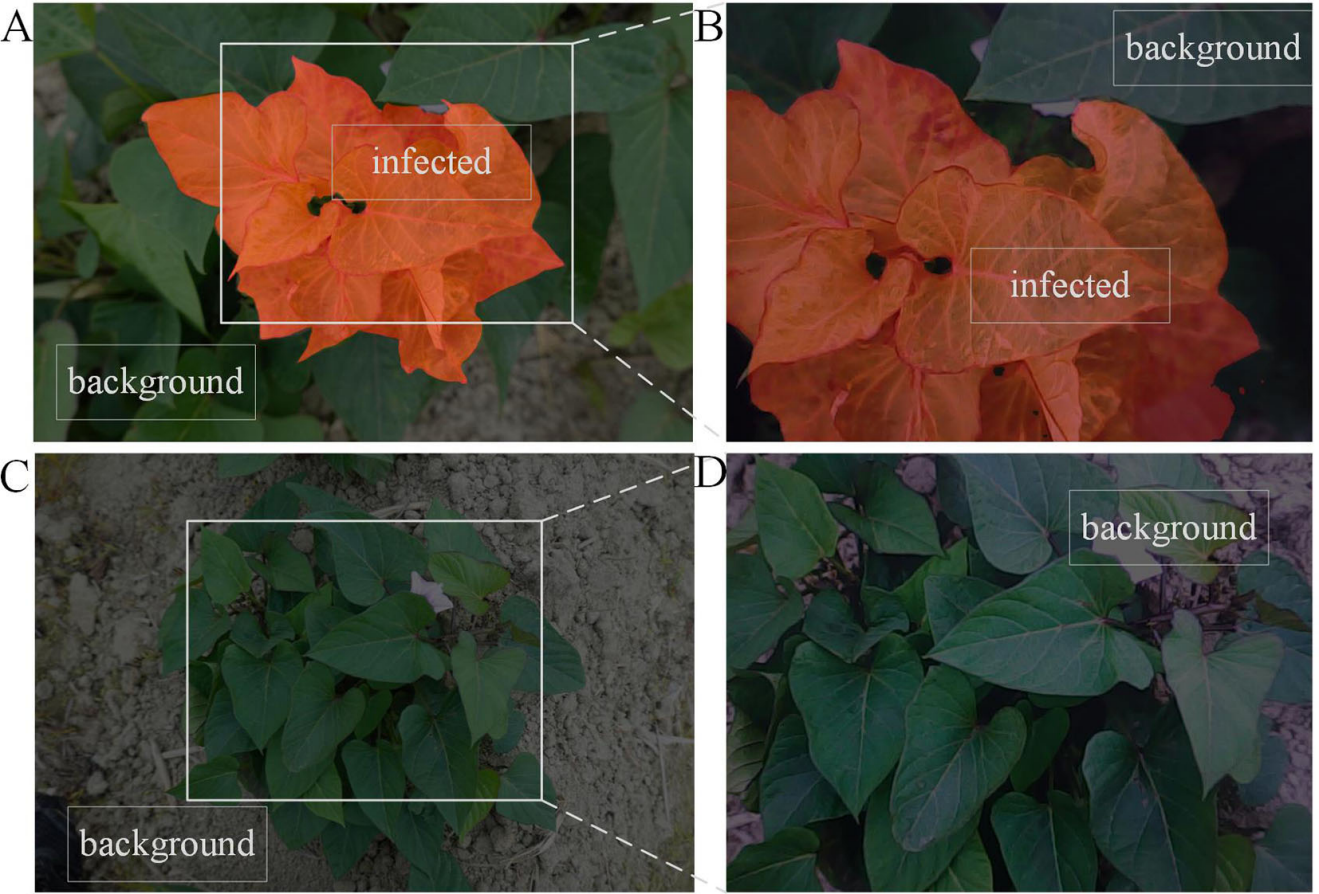
Model application verification under simulation scenarios. **(A, C)** Original images with manually annotation **(B, D)** Verification of the SPVD recognition effect of the model.

Using images collected from the field to simulate real-world scenarios, and **Figure 11** demonstrates the SPVD detection performance of the model. In **Figure 11A**, labeled as Sample 1, the manually annotated original image shows diseased areas marked in red, with the background consisting of healthy leaves and soil. The white solid line indicates the 480p camera’s capture window with a resolution of 480×640 pixels. **Figure 11B** shows the inference results from the camera frame within the capture window of **Figure 11A**, where the model accurately identifies the diseased leaves, effectively distinguishing them from the background.

In **Figure 11C**, labeled as Sample 2, the manually annotated original image includes no diseased leaves, and the background consists of healthy leaves and soil. **Figure 11D** shows the inference results from the camera frame within the capture window of **Figure 11C**, where the model accurately avoids misclassifying the healthy leaves in the background as diseased leaves.

## 4 Discussion

Computer vision and machine learning techniques have gained significant traction in agricultural applications, particularly in plant disease detection. However, despite advancements across various crops, there remains a lack of studies specifically addressing sweetpotato virus disease (SPVD). Considering sweetpotato’s global significance as a food crop and the severe economic impact of SPVD, this study evaluates the effectiveness of the DeepLabV3+ algorithm in accurately identifying SPVD infections on sweetpotato leaves. The work addresses a critical need for rapid, precise disease detection to support crucial crop management and disease control decisions.

A common challenge in processing large images for segmentation tasks is the computational limitations that often require either down-sampling or splitting the images into smaller segments. While down-sampling can reduce segmentation accuracy, splitting images can introduce irrelevant background content, leading to data inefficiencies. Researchers such as Chang et al. and Jiang et al. have emphasized the importance of dividing images into smaller segments to address this issue (Chang et al., 2024; Jiang et al., 2024). In this study, the latter approach was adopted, with a focus on optimizing the segmentation process and minimizing the influence of irrelevant background areas.

To simulate real-world agricultural conditions, a cost-effective 480p Guke camera with a resolution of 640×480 pixels was used. This camera reflects the types of equipment that farmers are likely to have access to, offering a practical scenario for evaluating the model. Additionally, the lower resolution provides an opportunity to test the model’s robustness in handling low-quality images, which is crucial in field conditions where high-resolution equipment may not always be available.

The DeepLabV3+ model was selected for its ability to capture both global and local features through atrous spatial pyramid pooling (ASPP). While the baseline model performed well, it encountered limitations when processing pure background images, occasionally failing to distinguish between diseased and healthy areas. To mitigate this, attention mechanisms and data augmentation techniques were introduced, significantly enhancing the model’s capacity to accurately segment diseased regions while reducing errors in background segmentation. Similar approaches have been noted by researchers, who also highlighted the effectiveness of these methods in improving segmentation performance (Qin and Hu, 2020; Yang et al., 2022).

In the ablation study, the baseline model combined with Attention Pyramid Fusion (APF) and Transfer Learning (TL) demonstrated the highest accuracy on the DS-1 test set. However, since the DS-1 dataset mainly focuses on diseased areas with limited background images, the model’s performance on pure background images was less satisfactory, occasionally resulting in misclassifications as square red areas. By adding data augmentation (DA), the model’s handling of pure background images improved, providing clearer segmentation and better overall performance, despite a slight reduction in accuracy.

While our improved model achieves good segmentation accuracy, there is still room for improvement, particularly in reducing segmentation time and parameter counts. Although the improved model performs slightly worse on DS-2 compared to DS-1, it still effectively recognizes similar disease features in other environments. For disease appearances not included in the DS-1 training set, particularly the purple diseased apical leaves, detection performance remains an area for improvement. Given the significant differences between various sweetpotato varieties, this result is not surprising and further emphasizes that future efforts could focus on expanding the dataset to include more growth stages and diverse varieties (with varying leaf colors and shapes between top and mature leaves), thereby enhancing the model’s generalization capability. Finally, validating the model under real field conditions will provide a comprehensive evaluation of its practical applicability.

## 5 Conclusion

By leveraging transfer learning, the improved DeepLabV3+ network’s accuracy is significantly enhanced. The incorporation of Attention Pyramid Fusion (APF) improves semantic feature representation, while the novel data augmentation technique increases generalization, allowing the model to handle background noise more effectively in large, real-world images. Achieving strong segmentation performance on both DS-1 (mIoU: 94.63%, mAcc: 96.99%) and DS-2 (mIoU: 78.59%, mAcc: 79.47%) datasets, the model also demonstrates robust detection in simulated field conditions. This solution offers a practical approach to SPVD identification, reducing the need for specialized expertise and providing farmers with an accessible, efficient detection tool.

## Data Availability

The raw data supporting the conclusions of this article will be made available by the authors, without undue reservation.
